# A hybrid deep learning framework combining transformer and logistic regression models for automatic marine mucilage detection using sentinel-1 SAR data: A case study in Armutlu-Zeytinbağı, Marmara Sea

**DOI:** 10.1371/journal.pone.0330721

**Published:** 2025-09-25

**Authors:** Enes Bakis, Emrullah Acar, Musa Yilmaz

**Affiliations:** 1 Electrical and Electronics Engineering Department, University of Piri Reis, İstanbul, Istanbul, Turkey; 2 Electrical and Electronics Engineering Department, University of Batman, Batman, Turkey; 3 Bourns College of Engineering, Center for Environmental Research and Technology, University of California at Riverside, Riverside, California, United States of America; Agricultural Sciences and Natural Resources University of Khuzestan, IRAN, ISLAMIC REPUBLIC OF

## Abstract

The identification of various objects and species found in nature is of great importance today. Active and passive imaging systems are in a beneficial position in this direction, both in terms of cost and convenience. Recently, mucilage events in our country pose a great risk for both marine life and human life. In this study, water areas in one of the regions affected by the mucilage event that occurred in May 2021 were chosen as the object. The region between Armutlu-Zeytinbağı in the Marmara Sea was chosen as the study area. 1300 samples were selected from the mucilage region and recorded with the help of GPS. After these selected samples were chosen as mucilage area for 17 May–22 May and as a clean area for 21 June-22 June (2600 samples in total), image analyses were made using time series with the help of Sentinel-1 satellite images. These image analyses were performed using Sentinel-1 band parameters (VV-VH). A unique data set was created by recording the numerical data showing the backscattering values of the VV-VH polarization band images. It is aimed to automatically detect the mucilage area by applying deep learning and machine learning to the obtained data set. It has been observed that the accuracies of our applied hybrid (Transformer Method + Logistic Regression), deep learning (RNN, CNN) and machine learning models (Decision Tree, Naive Bayes, SVM) are high (96%−100%). With the applied deep learning and machine learning methods, it is thought that regions can be detected more easily and intervened early in these regions.

## 1. Introduction

The mucilage problem in the Sea of Marmara first appeared in October 2007. The formation that fishermen call “sea saliva” creates an obstacle to the fishing of fishery products due to its dense structure. In addition, the mucilage event seriously damages the species diversity in the Marmara Sea [1]. It is seen that the mucilage incident reached serious levels in May 2021. In this context, early detection of mucilage areas is important both temporally and economically for events such as marine life and the sustainability of fishing activities. Identification of various objects and species in nature, automatic detection and extraction of the properties of these objects are of great importance today. Active and passive imaging systems are in a beneficial position in this direction, both in terms of cost and convenience. The Sentinel-1 satellite is also an active imaging system instrument. With the use of deep learning and machine learning techniques together with remote sensing systems, automatic detection of remote objects has become very easy today. Moreover, the detection of objects containing a large amount of data in many areas has been greatly facilitated thanks to these systems. In addition, deep learning and machine learning methods have an effective position in terms of comparing the accuracy and success rates of these data as a result of modelling.

Some of the studies on object recognition, mucilage area detection, object identification using remote sensing data and different machine learning techniques in the literature are as follows; Abaci et al. (2022) analyzed Sentinel-2 multispectral data and PRISMA hyperspectral data to detect mucilage at an early stage using both traditional and deep learning algorithms. They have shown that it is possible to detect mucilage with high accuracy from satellite data, saving time and money for cleaning work [2]. Kavzoğlu et al. (2021) aimed to identify the intensely observed mucilage formations around the Marmara Sea by focusing on the coasts of Istanbul, Kocaeli, Yalova and Bursa, using a new object-based image analysis (OBIA) approach paradigm by classifying the Sentinel-2A images dated 19 and 24 May 2021, where mucilage was most concentrated [3]. Acar et al. (2021) developed a methodology for mucilage detection by considering the current mucilage formation in the Sea of Marmara with the help of remote sensing (UA) techniques. For this purpose, mucilage formation from 10.03.2021 to 06.06.2021 were determined by classifying Sentinel-2 (MSI) satellite images with Google’s Earth Engine (GEE) platform and Random Forest (RF) algorithm [4]. Tassan (1993) established an algorithm that uses AVHRR data to detect a white tide from algal bloom (“mucilage”), an event that occurs under certain meteorological conditions in the Adriatic Sea. He then tested and mapped them [5]. Kavzoglu & Goral (2022) used five water indices estimated from cloudless and partly cloudy Sentinel-2 images acquired between May and July 2021 to effectively map mucilage clusters on the sea surface in the Gulf of Izmit using the cloud-based Google Earth Engine (GEE). They observed that mucilage aggregates, which started with covering approximately 6 km² of sea surface in ta, reached the highest level on May 24 and decreased at the end of July [6]. Tuzcu Kokal et al. (2022) presented the spectral properties of different mucilage species in the Gulf of Izmit in the Marmara Sea using medium (Sentinel-2) and high spatial resolution (Worldview-3) satellite images. The support vector machine (SVM) classifier was used to detect three different mucilage types with distinguishable spectral differences in the infrared region ranging from 725 to 950 nm [7]. Acar (2020) tried to detect the EDTs on agricultural lands in Diyarbakir by using the Sentinel-1 SAR satellite data by machine learning method. He used ELM (Extreme Machine Learning) in his study and achieved an accuracy rate of 85.47% [8]. Ertürk & Erten (2023) propose the use of blending on data sets acquired using the PRISMA satellite to analyze the spectral properties, aggregation-related variations and spatial distribution of marine mucilage. In their proposed approach, they showed that the abundance fraction maps obtained without a training step and using blending are easy to interpret and analyze for mucilage aggregation [9]. Colkesen et al. (2023) proposed a new spectral index, called the Automatic Mucilage Extraction Index (AMEI), which enables efficient and accurate detection of surface mucilage aggregates using Sentinel-2 satellite imagery. Their proposed index includes four bands (Bands 3, 4, 8 and 12) of Sentinel-2 Level-2A images covering the visible, near infrared and shortwave infrared regions. The index was formulated by taking into account the image obtained on 19 May 2021, when the most intense mucilage formations were observed in the Marmara Sea. The results confirm the robustness of the proposed spectral index, which offers superior separation performance (above 1.5 in terms of M-Statistic) compared to other water indices in both cloud-free images and images containing cumulus clouds. Visual interpretation also confirmed that the boundaries of mucilage formations in cloud-free and thin-cloud images are accurately defined by the proposed index and that different mucilage types (i.e., yellow and white) can be identified when an appropriate histogram thresholding is applied [10]. Sanver & Yesildirek (2023) aimed to design an autonomous marine mucilage monitoring system for systems such as unmanned surface vehicles (USV). The on-board solution they proposed in their study has an architecture based on a three-tier mucilage monitoring system. In the first stage, the sea surface is scanned in real time with camera(s) at a certain radius. When mucilage candidate areas are detected, the vehicle is autonomously guided to this area. In the second stage, the seawater in the area is measured in real time with some onboard sensors, pH level, conductivity and dissolved oxygen level. The third stage is where real samples are collected from three different depths (if possible) for detailed back-lab analysis. They compared image processing, CNN (ResNet50), kNN, SVM and FFNN approaches and showed that the accuracy of their proposed method is better and offers a more promising performance [11]. Messager et al. (2023) studied the use of SAR (Synthetic Aperture Radar) and Artificial Intelligence for ocean monitoring. For example, CMOD5.N (C-band MODel), a SAR model, was used in this study because it can provide wind speed estimates up to 25 m.s-1 with a deviation of 1–2 m.s-1 depending on the spatial wind grid and wind intensity. In addition, they have also conducted experiments on object detection (e.g., ships), sea ice and oil spills with successful results [12]. Sefercik et al. (2024) conducted a detailed study to evaluate the effects of mucilage phenomenon on seawater quality, sea surface temperature and backscattered radar signal strength in two different mucilage covered areas in the Sea of Marmara. Physico-chemical parameters such as sea surface temperature, electrical conductivity, hydrogen potential, suspended solids, dissolved oxygen concentration and chlorophyll-a were calculated from the water samples and the quality of mucilage-covered seawater was investigated. They also investigated the effects of mucilage on spectral reflectance, radar signal backscattering and sea surface temperature depending on the density of mucilage using space-borne synthetic aperture radar (SAR) and optical images of Sentinel-1, Sentinel-2 and Sentinel-3. In terms of sea surface temperature, the differences between clean seawater and medium-dense and dense mucilage areas were estimated to be 1.05–2.25°C, respectively [13].

This study is derived from a thesis published as a master’s thesis, the author of which is Enes BAKIŞ. Different from the thesis, the applied deep learning models were strengthened, extra layers were added and the success rate was increased by changing the number of epochs.

In this study, time series of Sentinel-1 satellite images have been utilized at a total of 1300 points that we manually marked the mucilage event that occurred in the Armutlu-ZeytinBağı region of the Marmara Sea on May 14–24, 2021. The detectability of these mucilage areas has been investigated by various applying deep learning and machine learning models to the backscattering values obtained with different bands (VV-VH) on the satellite. The success rate of deep learning and machine learning models applied in this field was found to be very high. This is important in order to distinguish the mucilage regions easily. In addition, the fact that the points are taken over the same region on different dates, but the data are distinguishable from each other, greatly contributes to this success rate. With the original data set created in the proposed study, it is aimed to automatically detect mucilage areas, prevent marine pollution and intervene in this pollution at an early stage. The research gaps of our study are presented in Table 1.

**Table 1:** The research gaps of our study.

No	Research Gap	Explanation
1	Limited use of Transformer models for mucilage detection with SAR data	Due to the structural complexity of SAR data, Transformer-based analyses are still scarce in the literature.
2	Underutilization of hybrid deep learning + logistic regression models in environmental classification	Despite the interpretability of logistic regression, such hybrid approaches are rarely used for mucilage detection.
3	Lack of localized mucilage detection studies in areas like Armutlu-Zeytinbağı	Existing studies mostly focus on the broader Marmara region, with limited attention to specific local areas.
4	Lack of model transparency in distinguishing mucilage in SAR imagery	The ability of models to separate mucilage from other surface elements in SAR images is not well explained.
5	Absence of temporal (time-series) analyses	Most current studies rely on single-date images; the temporal evolution of mucilage is not modeled.

The main contributions of our proposed study to the literature are as follows;

- Obtaining very high accuracy values as a result of the study (96%−100%),
- After automatically detecting mucilage areas, prevention of situations such as death of sea creatures and visual pollution with early intervention,
- To present the accuracy of the data and the success of automatic detection by using both deep learning and machine learning simultaneously with 2 pieces of data for the detection of mucilage areas,
- To minimize the mucilage level by automatically detecting the time intervals when mucilage occurs most often and intervening in these areas before time. In this study, May 2021 was accepted as the most common time of mucilage in the Marmara region. Therefore, the study was conducted for this time period. For later periods, it is aimed to detect this phenomenon automatically with instantaneous data and to minimize the possible damage by making the necessary interventions.

The rest of the manuscript is organized as follow. In Section 2, the study area, image processing, deep learning and machine learning models are given. In Section 3, the obtained backscattering values and the results of the applied learning models are given. According to the models used in Section 4, the success and usability of the system are discussed.

## 2. Materials and methodology

### 2.1. The experimental area

**Fig 1:**
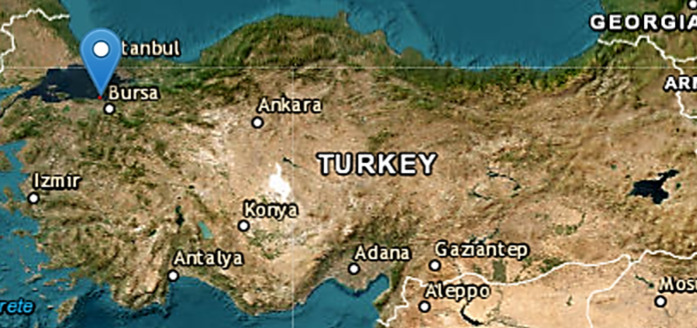
Position of the experimental area is indicated on USGS National Map Viewer (public domain). Here, the blue markers indicate the locations of selected 1300 point.

In this part, in the part between Armutlu and Zeytinbağı regions in the Marmara Sea, 1300 points for the mucilage area on 17 May and 22 May 2021, and the points selected for the mucilage area for the clean area on 21 June and 22 June were used again. Sentinel-1 (VV and VH polarizations) were used, along with GPS data collected at the selected points. The image of the mucilage area and the experimental area are given in **Fig 1**.

### 2.2. Sentinel-1 satellite data collection

In this phase, Sentinel-1 satellite images with VV and VH polarizations belonging to 4 different dates (May 17, May 22, June 21 and June 22, 2021) were employed with the aid of Google Earth Engine Code Editor (GEE-CE) Platform. (https://code.earthengine.google.com). Data was accessed through GEE-CE platform. In addition, the data set was limited to the Armutlu-Zeytinbağı region and only data from the time of mucilage (2021 May) were taken.

### 2.3. Object data collection

In this phase, the geographical coordinates of mucilage area and clean area with a total of 1300 samples were recorded with the help of GEE-CE platform and the recorded data were then imported to the Sentinel-1 data.

During this importing process, Google Earth Engine (GEE) was used to create points at certain coordinates (latitude and longitude) and assign properties to these points. For example, the steps for transferring two points were as follows:

- Point Definition: Two points were defined using the ee.Geometry.Point function. The coordinates of the first point were [28.80965094959788, 40.50079377807344]. The coordinates of the second point were [28.80967694675749, 40.500768257922154].
- Property Assignment: For each point, a property dictionary was defined. The “w (water)” property was set to 1 for both points. This one value indicated that the points start from 1 when the dataset was created. The property “system:index” was used to assign a unique identification number to each point and took the values “1” and “2” respectively.
- Creating ee.Feature: Each defined point and feature was saved as an ee.Feature object. Two feature points were created and specific features were assigned to each feature. These features can then be used for analysis, visualisation or other operations.

Here, the dates of the GPS data were not chosen randomly. Of course, mucilage formation is in a constantly moving structure. This raises the question of how GPS data is acquired. Specifically, GPS data were obtained from mucilage regions (Marmara Sea/Armutlu-Zeytinbagi) proven in previously published articles [14], between the dates when mucilage occurred. After these dates were determined, Sentinel-1 satellite data (VV-VH) were taken on these dates. The other question that comes to mind is that mucilage is constantly changing, but how was the data obtained on the same point on May 17 and May 22. The answer to this is that the mucilage problem that occurred in May 2021 is the biggest mucilage problem in the history of Turkey. These intense mucilage formations persisted for weeks, rather than just 2−3 days. The busiest period is between 14−22 May. These explanations seem to be supported by previously published articles. Therefore, the data obtained after 5 days over the same point again points to the mucilage area. In summary, GSP data were instantly transferred to Sentinel-1 satellite data on each point marked in the region where the formation of mucilage was located, and 2 data sets were obtained by taking the VV-VH band parameters and the backscattering values of these points in those dates.

All Sentinel-1 SAR data used in this study were accessed and processed via the Google Earth Engine Code Editor (GEE-CE) platform. The preprocessing workflow included:

- Filtering by polarization (VV, VH)
- Orbit correction and border noise removal
- Temporal filtering based on the bloom periods
- Speckle noise reduction and image compositing

The classification and hybrid model implementation were carried out in Python 3.10, using libraries such as TensorFlow, scikit-learn, and NumPy. Detailed algorithmic steps, including Transformer-based feature extraction and Logistic Regression classification, were described in the Methods section.

Although no public code repository is available at this time, all scripts and implementation details can be shared upon reasonable request to the corresponding author.

**Fig 2:**
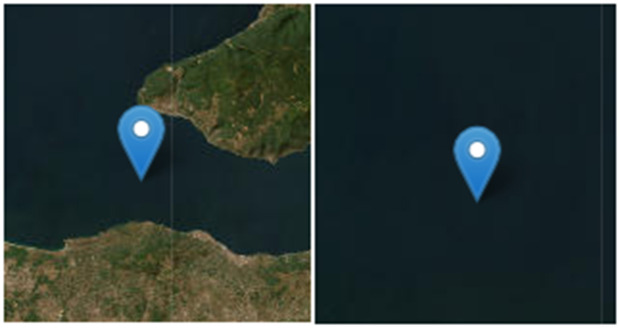
This image shows (a) the unzoomed version of the Sentinel-1 SAR image and (b) the zoomed version of the Sentinel-1 SAR image for a point in the selected experimental area. The image was acquired on 10 June 2024. Sentinel imagery was freely downloaded from the Gateway to Astronaut Photography of Earth (https://eol.jsc.nasa.gov/SearchPhotos/).

The image of how the mucilage appear on the Sentinel-1 Synthetic Aperture Radar is given in **Fig 2**.

### 2.4. Feature extraction

The Sentinel-1 mission ensures data from C-band Synthetic Aperture Radar (SAR) device at 5.405 GHz. This collection contains S1 Ground Range Detected scenes, which are calibrated, ortho-corrected product. Each scene has three resolutions (40, 25 and 10 meters), three instrument modes and four band combinations (corresponding to scene polarization) [15]. The pixel resolution of the satellite used in this study was 10 meters. The bands used were VV (vertical transmit & vertical receive) and VH (vertical transmit & horizontal receive). The reason for using VV-VH band parameters in this study was to take the backscattering values of the data to be obtained at each marked point. HV and HH bands hadn’t been preferred because they send horizontal signals. Correlation analysis was performed for the dataset in which VV and VH data sets were used together. This analysis results were presented in Table 2. In addition, the Sentinel-1 satellite employs a transmission and reception of electromagnetic signals (microwaves) to perform its functions. The signal values received are referred to as backscatter values. This is the reason why it is referred to as a Synthetic Aperture Radar, and it enables the acquisition of images with an extremely high level of resolution.

**Table 2:** The Correlations values (%) for VV + VH data set.

	May17(M)-Jun22(T) [VV]	May22(M)-Jun21(T) [VV]
May17(M)-June21(T) [VH]	85.4%	95.9%
May22(M)-June22(T) [VH]	86.1%	97.3%

Table 2 confirms that the correlation values in the VV + VH dataset are high.

### 2.5. Obtaining Dataset

**Fig 3:**
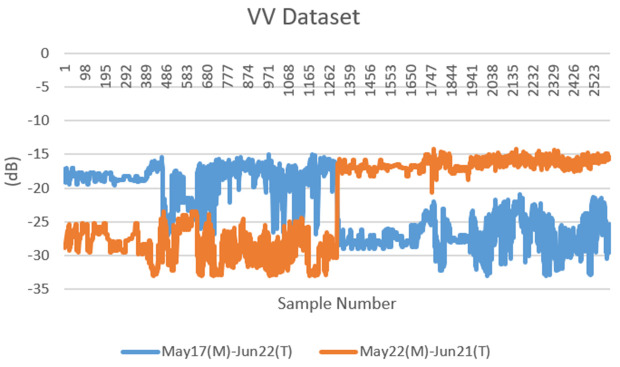
Distributions of VV datasets.

**Fig 4:**
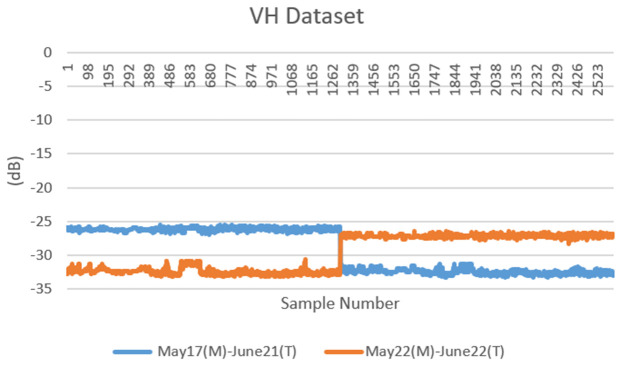
Distributions of VH datasets.

**Fig 5:**
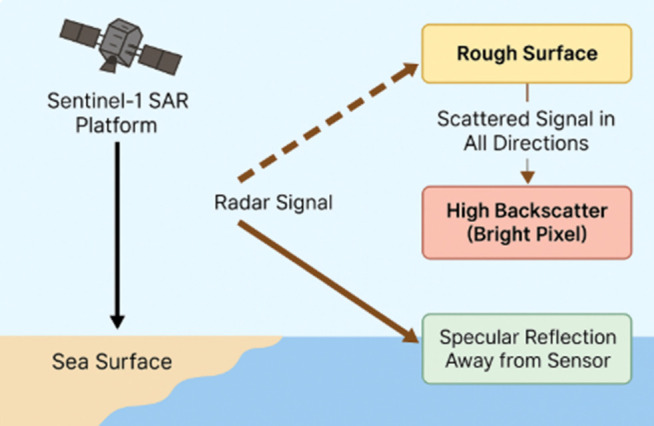
The SAR backscattering response to surface roughness and dielectric properties.

The dataset consists of backscatter values obtained at 1300 points marked on the same region on different dates with the help of Sentinel-1 satellite band parameters (VV-VH). The dataset is formed by combining the backscatter values of the mucilage area (17 and 22 May) and clean area (21 and 22 June) for 1300 points. In other words, two datasets containing a total of 1300 samples, 4 features and 2 classes were created for both VV and VH band parameters. These two datasets were combined to create a dataset with 2600 samples. The obtained datasets were then applied to deep learning and machine learning models separately. The dataset created for both VV and VH band parameters was useful for comparing the application results. Furthermore, the congruence of the selected points, despite the disparity in dates, facilitated a more precise examination of the mucilage area within its respective region. The datasets are given in Table 3 for VV band and Table 4 for VH band. The data distributions of the datasets are shown in Fig 3 and Fig 4, respectively. The SAR backscattering response to surface roughness and dielectric properties is shown in **Fig 5**.

**Table 3:** Generated dataset for VV band.

Sample Number	May17(M)-Jun22(T) [VV]	May22(M)-Jun21(T) [VV]	Class
0	−19.005	−28.474	1
1	−19.005	−28.474	1
2	−17.107	−28.544	1
3	−17.107	−28.544	1
4	−17.928	−28.952	1
…	…	…	…
2595	−26.864	−15.690	0
2596	−25.752	−15.353	0
2597	−29.599	−15.690	0
2598	−26.864	−15.690	0
2599	−25.400	−15.465	0

**Table 4:** Generated dataset for VH band.

Sample Number	May17(M)-Jun21(T) [VH]	May22(M)-Jun22(T) [VH]	Class
0	−25.927	−32.774	1
1	−25.927	−32.774	1
2	−26.232	−32.395	1
3	−26.232	−32.395	1
4	−26.928	−32.343	1
…	…	…	…
2595	−32.866	−26.981	0
2596	−32.910	−27.116	0
2597	−32.839	−26.981	0
2598	−32.866	−26.981	0
2599	−33.073	−26.903	0

As seen in Tables 3 and 4, the datasets have 4 columns and 2600 rows. The first column contains 2600 data samples. 1300 of these samples contain the data obtained for the mucilage water area, and the other 1300 samples contain the data for the clean water area. The second and third columns include the dates of data collection. Data set contains mucilage field data for May 17 in column 2 and May 21 in column 3 as well as fresh water area data for 21 June in column 2 and June 22 in column 3. This data is assigned a class value in the fourth column. The reserved class for the mucilage area is ‘0’, the reserved class for the clean area is ‘1’.

The unit of numerical values (y-axis) in Fig 3 and Fig 4 for the VV and VH band of the Sentinel-1 satellite is expressed in “dB” (decibels). This is a logarithmic measure of the amount backscattered. Sentinel-1 data measures the backscattered power of radar signals and reports this power in decibels. The decibel scale is a logarithmic measure of the strength or intensity of a signal.

The values in second and third columns are attribute values and represent the backscatter values at each point. The backscatter values obtained from the Sentinel-1 satellite can be represented as a negative number. This means that part of the reflected signal is absorbed or scattered by reflective surfaces before reaching the receiver. This can occur especially on surfaces with non-reflective properties (e.g., water, asphalt, etc.) or in areas with low reflectivity. The algorithms used to obtain these values can be briefly explained as follows:

1. Filtering image collections containing VV and VH polarised backscatter data from Sentinel-1 satellite on a specific date (between 2021-05-17 and 2021-05-23).
2. Using the vector data containing a specific region (water), time series plots are created to visualise the backscatter values for that region.
3. The graphs show time on the horizontal axis and mean backscatter values on the vertical axis. Creation of separate graphs for VV (vertical polarisation) and VH (horizontal polarisation).
4. The graphs show red colour for cities, green colour for forests and blue colour for deserts. These colours represent land types with different uses.

The applied deep learning models (CNN, RNN) and machine learning models (SVM, Decision Tree, Naive Bayes) have been implemented on Jupiter Notebook using python 3.0 version.

### 2.6. CNN architecture

CNN is often used for image analysis. It is a successful method in classifications. The basis of the CNN model, which is organized in layers, is based on neurons. Each neuron in these layers is connected by weights and biases. Therefore, the basis of CNN’s architecture is based on the change of these weights. In a cycle, the weights change until they reach the correct value. CNN model has input layer, convolution layer, pooling layer, full connection layer, output layer [16].

In this study, a sequential model was employed for the CNN model instead of ready architecture. In the proposed model, 3 convolution layers, 2 pooling layers and 2 dense layers were used and the “man” function was chosen for optimization. Since our class has 2 units, the “sigmoid” activation function was utilized in the output layer, and “ReLu” was preferred for the activation function in other layers. Moreover, the ‘’binary cross entropy” structure was employed to find the missing values, and the “accuracy” structure was used to find the correct values. The principal rationale for adopting a three-layer structure in the convolutional neural network (CNN) model is the optimal depth for learning and analyzing the data set. The rationale for limiting the number of layers is to avoid the potential issue of overlearning the model. In consideration of the data set and the analysis, a depth of three layers is deemed sufficient.

**Fig 6:**
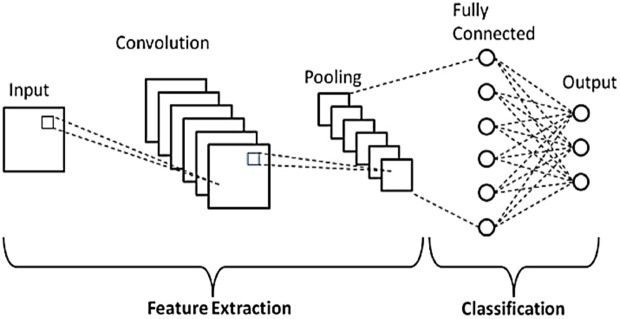
CNN architecture [17].

CNN Architecture is shown in **Fig 6**.

### 2.7. RNN architecture

In the RNN (Recurrent Neural Network) architecture, the information coming to the neurons in the layers tries to create a prediction by multiplying certain weight values. When this estimate reaches the true value, the weights are changed and multiplied again. In other words, RNN is architecturally similar to CNN architecture. The main difference between a CNN and an RNN is its ability to process temporal information. While RNN is designed for this purpose, CNN cannot process temporal information as effectively as RNN [18].

In the proposed work, sequential model was utilized for the RNN model. In the model, three LSTM (Long Short-Term Memory) layers and 2 dense layers were employed. The ‘man’ function was chosen for optimization. In the output layer, the “sigmoid” activation function was and “ReLu” was preferred for the activation function in other layers. Moreover, the “binary cross entropy” structure was used to find the missing values and the “accuracy” structure was used to find the correct values. The rationale for employing two layers in the RNN model is that it possesses sufficient depth to facilitate the analysis and learning of dependencies in both short- and medium-term situations.

**Fig 7:**
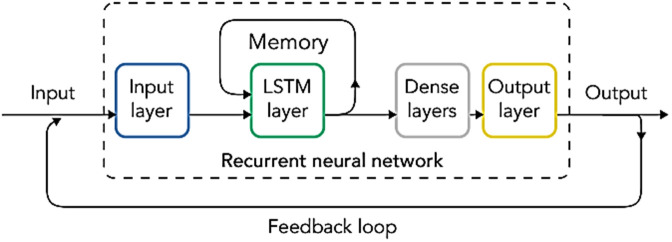
The working principle of RNN [19].

The working principle of RNN is shown in **Fig 7**.

### 2.8. SVM architecture

**Fig 8:**
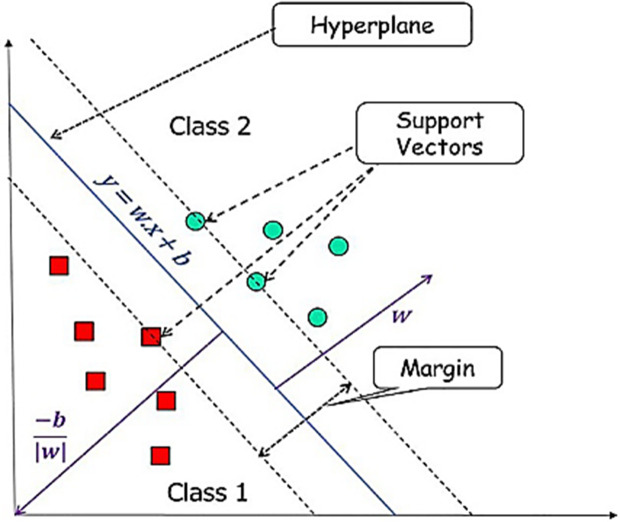
The working principle of SWM [21].

SVM (Support Vector Machine) is one of the machine learning methods used especially in classification problems. Draws a line segment to distinguish between points placed on a plane. It aims to have this line at the maximum distance for the points of both classes. This machine learning method is suitable for complex but small and medium sized datasets [20]. This model was selected for its capacity to achieve high accuracy, due to its ability to effectively separate the data by accurately learning the classification boundaries. The working principle of SWM is shown in **Fig 8**.

### 2.9. Decision tree (DT) architecture

**Fig 9:**
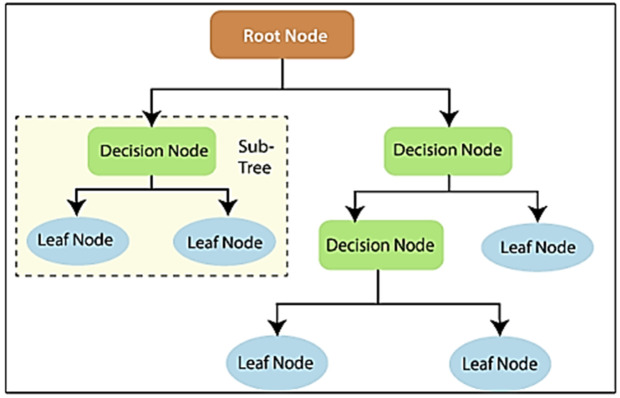
The working principle of DT [23].

The decision tree model is often used in Classification and Regression problems. The first cell in decision trees is the stem cell. Each situation is classified as yes or no depending on the conditions specified in the root. Below these stem cells are intermediate roots (interval roots or nodes). Each possible situation is classified with the help of these intermediate roots. This model has leaves at the very bottom. It is the part that shows the result of the classification [22]. The decision tree model is particularly adept at discerning the layered structure of data, particularly in non-linear datasets. This quality was a primary factor in its selection, as well as for comparison with other models. The working principle of DT is shown in **Fig 9**.

### 2.10. Naive bayes (NB) architecture

**Fig 10:**
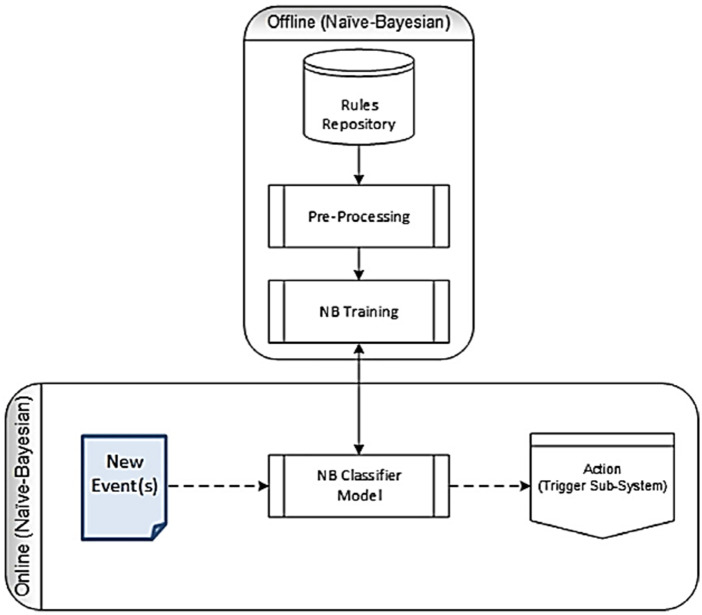
The working principle of Naive Bayes [25].

Naive Bayes method is based on Bayes theorem. The working principle of the algorithm calculates the probability value for each element and classifies according to the highest probability value. If the value in the test set does not correspond to a value in the training set, the probability is taken as 0. This situation is called zero frequency [24]. This model was selected for comparison with other models due to its rapid processing speed and straightforward architecture, which enables high accuracy, particularly when the data exhibits a clear separation between classes. The working principle of Naive Bayes is shown in **Fig 10**.

### 2.11. Hybrid model approach

**Fig 11:**
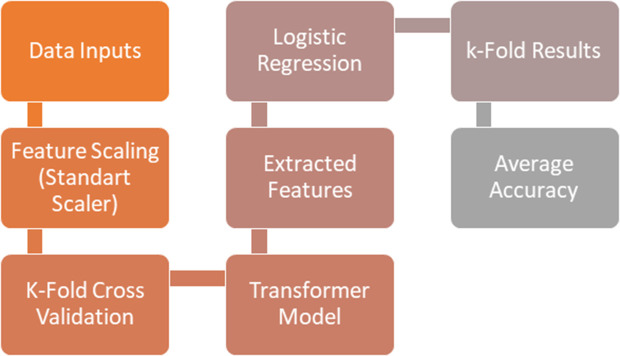
The working principle of Hybrid Model.

In this study, the Transformer model and the Logistic Regression model were employed in an integrated manner. Following the normalization of the data using the StandartScaler, a cross-validation process was employed to separate the data into a training and test set comprising five-fold cross-validation. Training was conducted for each transformer encoder layer. The Transformer Encoder Layer transforms the input data into a 128-dimensional feature vector, which was then processed by a multi-headed attention and feedforward network to learn the relationships between the features. The meaningful features derived from the trained system were subsequently fed into the Logistic Regression model for classification. Once the values of reliability calculated for each fold, the mean value was determined. In conclusion, the hybrid model constructed using Transformer’s deep learning capabilities and LR’s fundamental ability to perform classification with high accuracy, thus demonstrating the potential of combining these two techniques. The hybrid structure of this model was designed to enhance accuracy by leveraging the strengths of both models. While the Transformer is capable of learning more complex and contextual features of the data, the Logistic Regression was able to utilize this information in an efficient manner for the purpose of classification. It was therefore concluded that the hybrid model is the preferred option. The working principle of hybrid model is shown in **Fig 11**.

## 3. Experimental results and discussion

It is extremely important to use geographic information systems together with remote sensing techniques to determine the polarization band parameters of various objects. In this study, GEE-CE platform used as a geographic information system interface. It is possible to take both satellite and normal images of various regions via this platform. In addition, this interface has a coding system with a java infrastructure. Thanks to this coding system, satellite images can be taken in the desired date range after integrating various satellites with this platform. In this study, the backscattering values of the mucilage area on a certain date were obtained with the VV and VH band parameters of the Sentinel-1 satellite using the GEE-CE platform.

**Fig 12:**
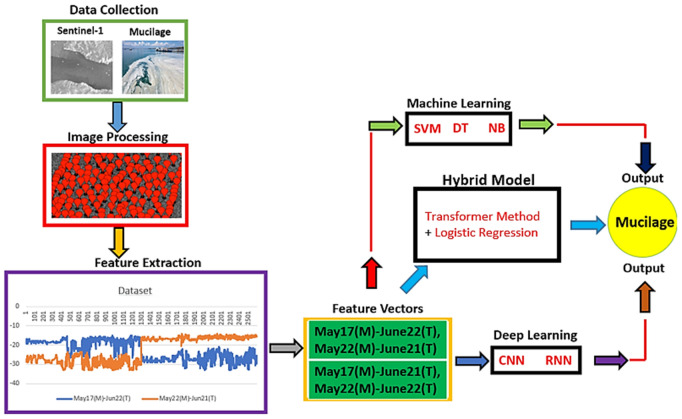
Work flow diagram.

The proposed work consists of several stages. First of all, the study area was determined as the water region between Armutlu-Zeytinbağı in the Marmara Sea of Turkey. In the second stage, 1300 points were selected manually at different times in this selected region. Then, the positions of these points were recorded by the GEE-CE platform and these GPS data were transferred to the Sentinel-1 satellite data. Image analyzes were performed to calculate polarization band parameters from Sentinel-1 data. In addition, it is aimed to automatically determine whether the region is mucilage or clean with the polarization band parameters obtained using both deep learning and machine learning approaches in addition to hybrid model. The block diagram of the proposed mucilage field detection system was given in **Fig 12**.

### 3.1. Statistical performance metrics

**Fig 13:**
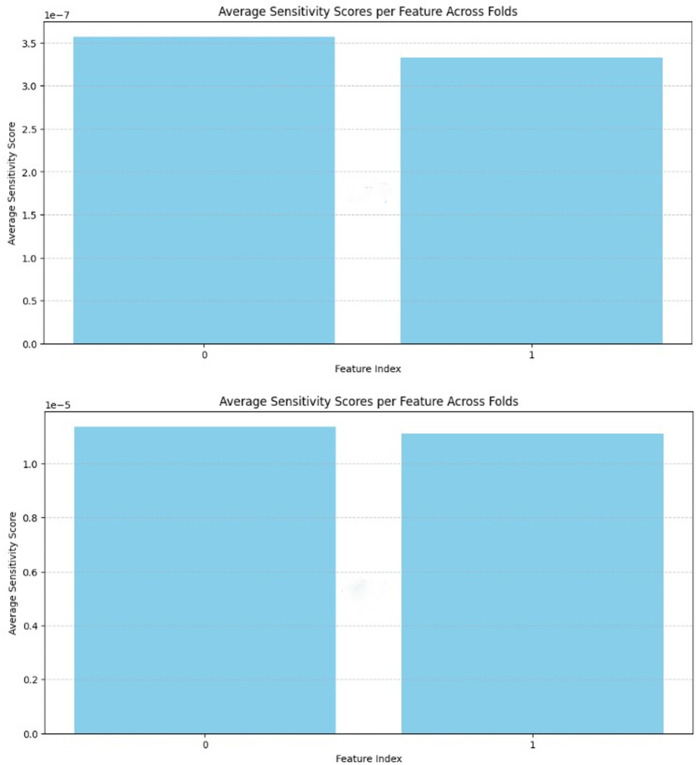
Sensitivity analyses using Hybrid model for both VV and VH bands.

In this section, some metrics were utilized in order to validate the results of the study statistically. These metrics were F-1 score, recall, precision, specificity, confusion matrix, ROC curve, accuracy-loss graphs, and a table with general results. Some calculations were made on the confusion matrix and transferred to the general table. In addition, sensitivity analyses were performed by making 10% change in the input data in the data set. As an example, sensitivity analyses using Hybrid model for both VV and VH bands are shown in **Fig 13**.

When Fig 13 was examined, it was observed that the performance of the system remained stable, although the input data was increased and decreased by 10% in sensitivity analyses. This demonstrated the stability of our data set and its resistance to alterations in the input parameters. Concurrently, it was established that the backscattering values of the mucilage in the mucilage region exhibited significant variation in comparison to those observed in a standard water environment.

The confusion matrix is shown in **Table 5**. The AUC value seen on the Roc curve shows us the accuracy of the result. This value ranges from 0 to 1. Similarly, the average accuracy rate of the study can be seen on the graphs. The general table includes all the results of the study. These tables and other metric methods used demonstrate the accuracy of the study.

**Table 5:** Confusion matrix structure.

		Predictive Values		
		0	1	**SUM**
**Actual Values**	0	TN	FP	TN + FP
	1	FN	TP	FN + TP
	**SUM**	TN + FN	FP + TP	TN + FN + TP + FP

The equations used in the calculations over the confusion matrix are given below.

- Accuracy (ACC) = $\frac{TP+TN}{TP+TN+FN+FP}$ : Indicates the accuracy value of the model.
- Sensitivity (TPR) = $\frac{TP}{TP+FN}\;$ : Indicates the sensitivity level of the model.
- Fall-Out (FPR) =$\;\frac{FP}{TN+FP}$: Indicates the false positive rate of the model.
- Miss Rate (FNR) =$\frac{FN}{TP+FN}$ : Indicates the false negative rate of the model.
- Specificity (TNR) =$\frac{TN}{TN+FP}$ : Indicates the true negative rate of the model.
- Precision (PPV) =$\frac{TP}{TP+FP}$ : Indicates the positive predicted value of the model.
- False Omission Rate (FOR) =$\frac{FN}{TN+FN}$ : Indicates the false omission rate of the model.
- False Discovery Rate (FDR) =$\frac{FP}{TN+FN}$ : Indicates the false discovery rate of the model.
- Negative Predictive Value (NPV) =$\frac{TN}{TN+FN}$ : Indicates the negative predicted value of the model.

**TN:** Total True Negative Values: Shows the total amount of data with a true value of ‘negative’ and predicted as ‘negative’.

**TP:** Total True Positive Values: Shows the total amount of data with a true value of ‘positive’ and predicted as ‘positive’.

**FN:** Total False Negative Values: Shows the total amount of data with a true value of ‘positive’ and predicted as ‘negative’.

**FP:** Total False Positive Values: Shows the total amount of data with a true value of ‘negative’ and predicted as ‘positive’.

### 3.2. VV polarization band results

**Fig 14:**
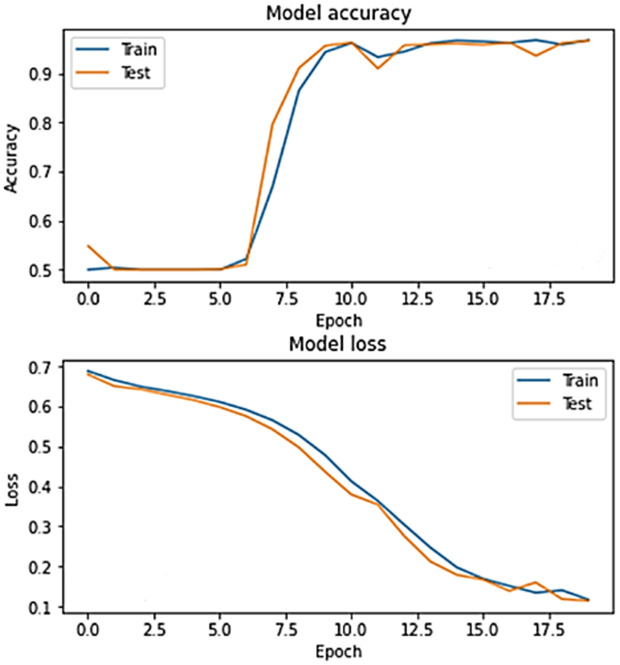
Accuracy and loss graphs of the training and test data of the VV band for the CNN model in the trained system.

**Fig 15:**
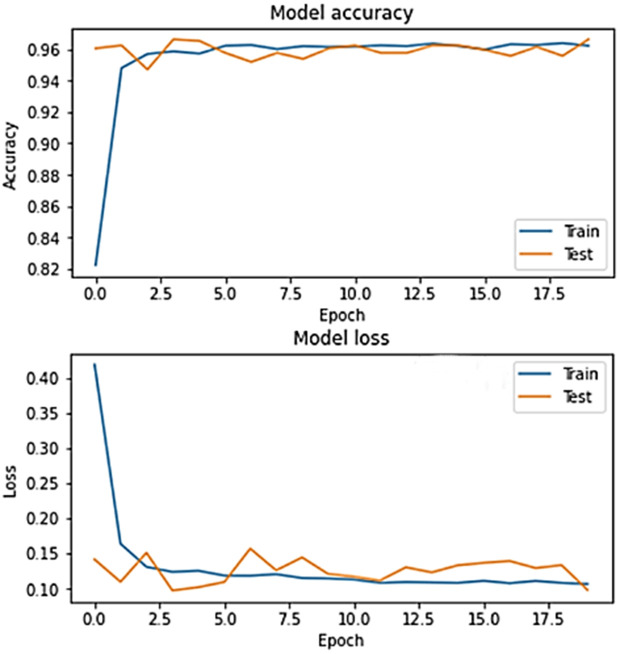
Accuracy and loss graphs of the training and test data of the VV band for the RNN model in the trained system.

**Fig 16:**
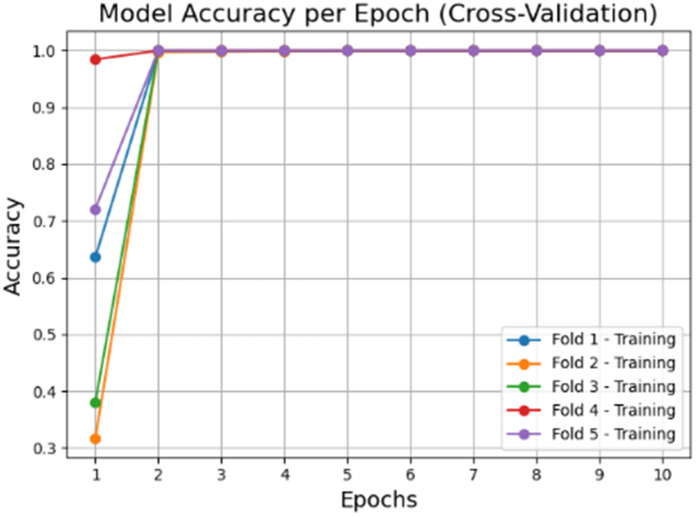
Average accuracy values of the hybrid model for the training set after cross-validation per epoch.

**Fig 17:**
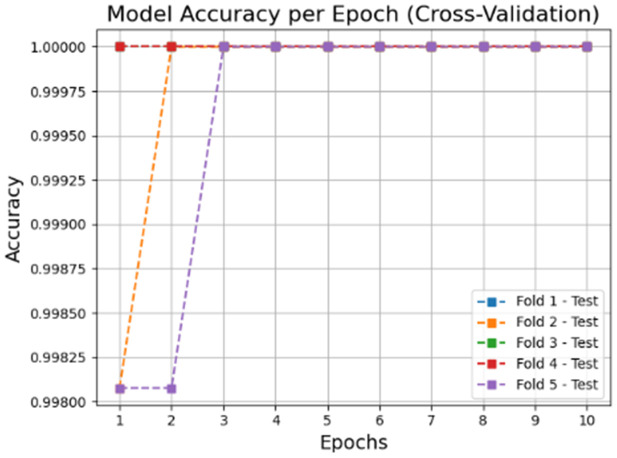
Average accuracy values of the hybrid model for the test set after cross-validation per epoch.

The accuracy and loss graphs in deep learning models of the dataset created by using VV polarization band for mucilage and clean water areas are shown in **Fig 14** and **Fig 15**, respectively. In addition, the average accuracy plots for each fold of the hybrid model are given in **Fig 16** and **Fig 17** for training and testing, respectively.

When the Figs 14–17 are examined, it is seen that the successes of both models are high (96%−100%) and the loss rates are low.

**Fig 18:**
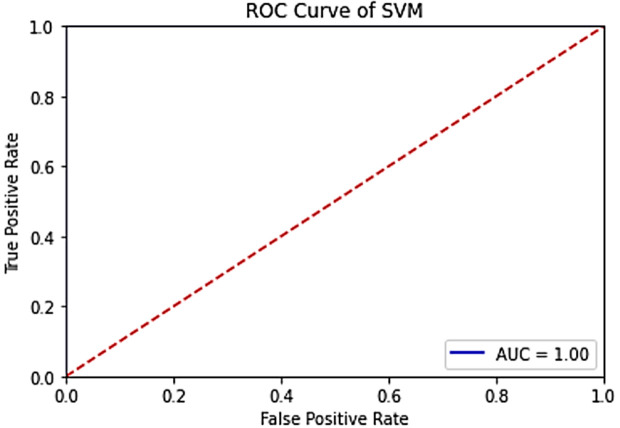
ROC curve of the VV band for the SVM model in the trained system.

**Fig 19:**
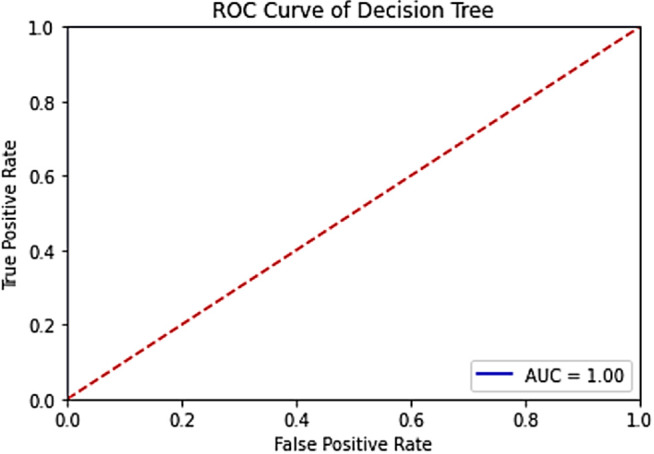
ROC curve of the VV band for the Decision tree model in the trained system.

**Fig 20:**
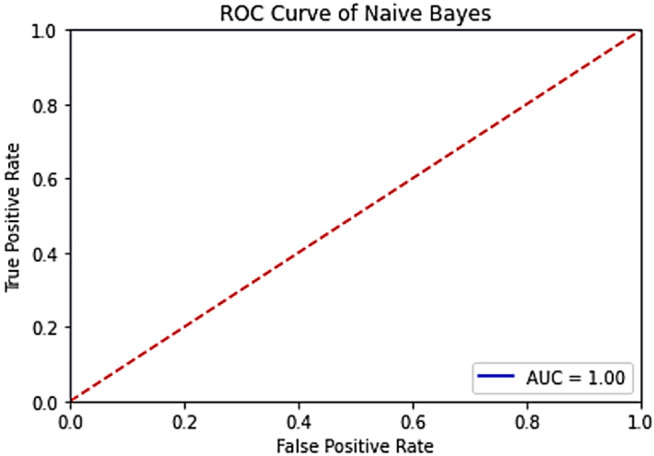
ROC curve of the VH band for the Naive Bayes model in the trained system.

The results of the machine learning models used (SVM, Decision Tree, Naive Bayes) are given in **Fig 18**, **Fig 19** and **Fig 20**, respectively.

When the Figs 18–20 are evaluated, the highest accuracy values (=100%) were obtained in all of the machine learning models used. This proves that the dataset contains accurate and distinguishable data.

### 3.3. VH polarization band results

**Fig 21:**
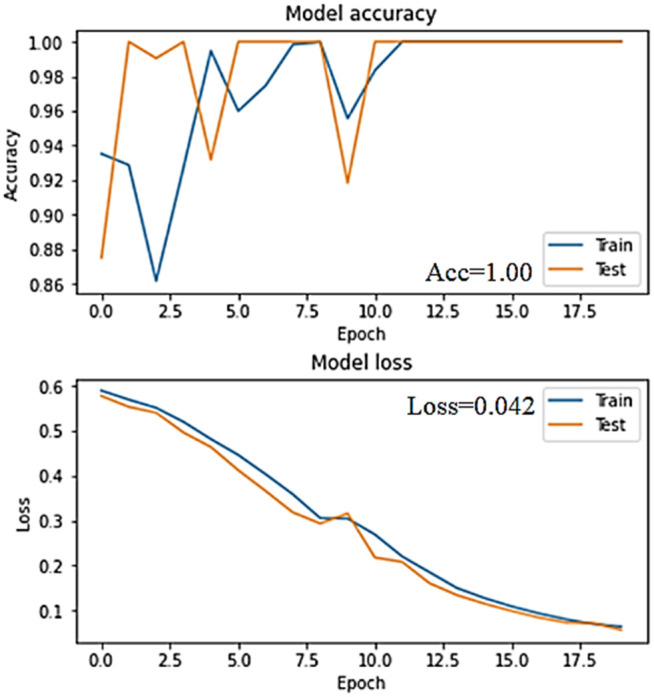
Accuracy and loss graphs of the training and test data of the VH band for the CNN model in the trained system.

**Fig 22:**
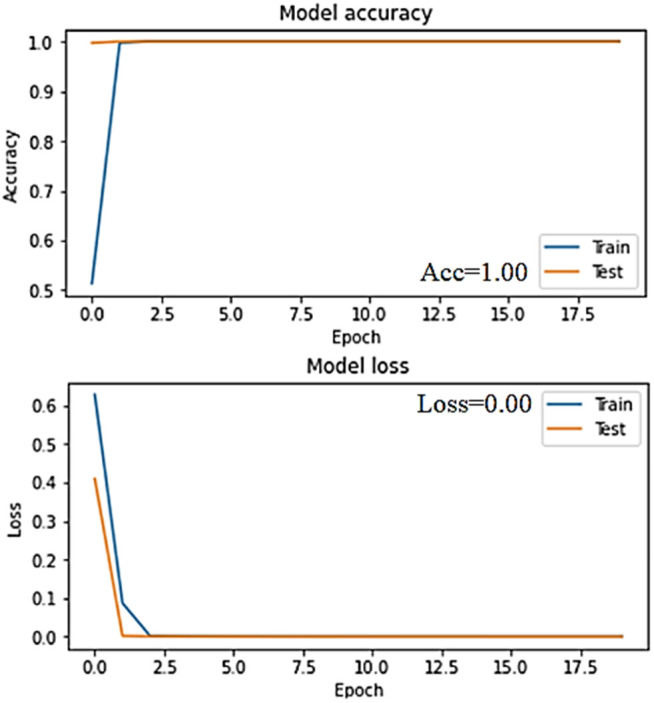
Accuracy and loss graphs of the training and test data of the VH band for the RNN model in the trained system.

**Fig 23:**
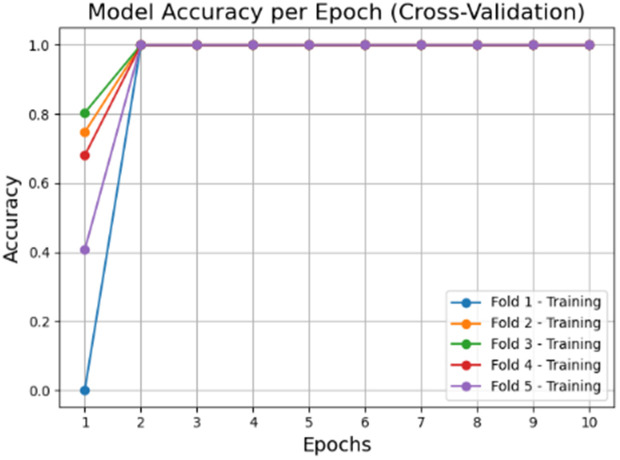
Average accuracy values of the hybrid model for the training set after cross-validation per epoch.

**Fig 24:**
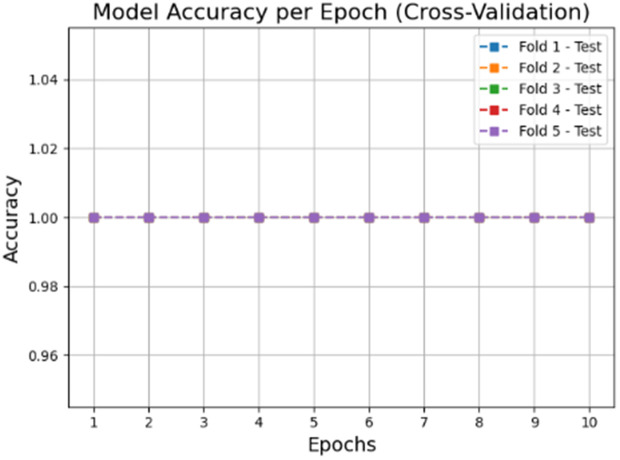
Average accuracy values of the hybrid model for the test set after cross-validation per epoch.

The accuracy and loss graphs in deep learning models of the dataset created by using VH polarization band for mucilage and clean water areas are shown in **Fig 21** and **Fig 22**, respectively. In addition, the average accuracy plots for each fold of the hybrid model are given in **Fig 23** and **Fig 24** for training and testing, respectively.

Considering the Figs 21,22,23 and 24, it is observed that the dataset created for the VH band parameter has the highest accuracy value (=100%) for all models.

**Fig 25:**
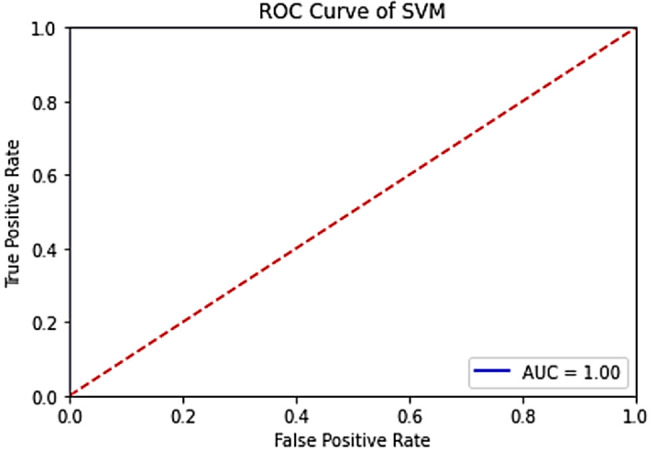
ROC curve of the VH band for the SVM model in the trained system.

**Fig 26:**
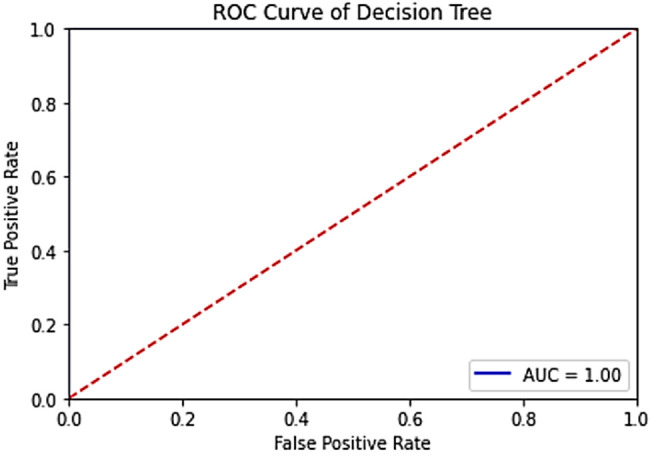
ROC curve of the VH band for the Decision Tree model in the trained system.

**Fig 27:**
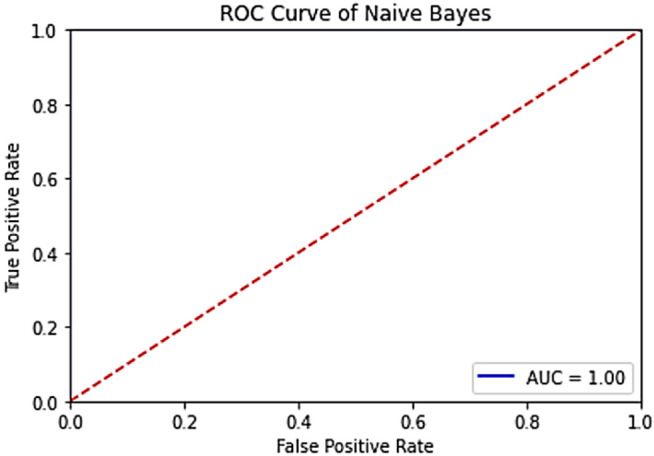
ROC curve of the VH band for the Naive Bayes model in the trained system.

The results of the machine learning models used in the dataset of the VH band (SVM, Decision Tree, and Naive Bayes) are given in **Fig 25**, **Fig 26** and **Fig 27**, respectively.

When the Figs 23–25 are examined, the highest accuracy values (=100%) were obtained in all of the machine learning models used. This proves that the dataset contains accurate and distinguishable data.

Figs 18–20, 25–27 show the ROC curves. The ROC curve has an important place in contributing to the study and showing the success of the models. Some advantages of the ROC curve are as follows;

- **Threshold-Independent Evaluation:** ROC curve allows evaluating model performance across a range of classification thresholds, not just at the default (e.g., 0.5), providing a more flexible analysis.
- **Balances True Positives and False Positives:** It simultaneously visualizes the True Positive Rate (TPR) and False Positive Rate (FPR), helping to understand the trade-off between detecting positive cases and minimizing false alarms.
- **Provides an Overall Summary via AUC:** The Area Under the Curve (AUC) gives a single scalar value that reflects the model’s ability to discriminate between classes, making model comparison straightforward.
- **Effective for Imbalanced Datasets:** Unlike accuracy, which can be misleading in imbalanced datasets, the ROC curve (and especially AUC) offers a more reliable measure of performance.
- **Enables Easy Visual Comparison:** By plotting multiple ROC curves in the same graph, researchers can visually compare the performance of different models or configurations at a glance.

When the confusion matrices in the Table 6 are evaluated, the success rate of all confusion matrices is 100%, except for the confusion matrix, which shows CNN and RNN model success for the VV band. This shows that the success rate of the VH band is maximum for all models and gives better results than the VV band. This may be due to the fact that the VH band receives data horizontally while receiving data. Because mucilage spreads in large areas, not pointwise. However, since almost the entire area was covered with mucilage at the time the data of the mucilage were taken, point (VV-VH) was preferred as data sending.

**Table 6:** The confusion matrices parameters belonging to models applied to datasets (VV and VH).

Model	Polarization Band	TN	FP	FN	TP
Hybrid	VV	386	1	1	392
	VH	387	0	0	393
CNN	VH	365	22	6	387
	VH	387	0	0	393
RNN	VV	362	25	5	388
	VH	387	0	0	393
SVM	VV	387	0	0	393
	VH	387	0	0	393
Naive Bayes	VV	387	0	0	393
	VH	387	0	0	393
Decision Tree	VV	387	0	0	393
	VH	387	0	0	393

**Table 7** shows that the success rate changes according to the number of layers selected for the CNN model for VV dataset as an example. This table emphasises why 3 layers are selected in the CNN model.

**Table 7:** Effects of Convolutional Layer Depth on Classification Performance for VV dataset.

CNN Structure	Accuracy	F1-Score	Epoch Time	Overfitting Status
2-layer	0.91	0.9	14s	Underfitting
3-layer	0.96	0.95	17s	Balanced
4-layer	0.95	0.94	24s	Beginning to overfit

**Table 8** contains statistical results data of all applied models. These data have been verified both on the confusion matrix and on the graphs and ROC curves given. **Table 9** shows the F-1 Score, Specificity, Recall and Precision metrics for all models.

**Table 8:** The statistical results data of all applied models.

Model	Polarization Band	ACC	TPR	FPR	FNR	TNR	PPV	FOR	FDR	NPV
Hybrid	VV	99%	99%	0,26%	0,25%	99%	99%	0,26%	0,26%	99%
	VH	100%	100%	0%	0%	100%	100%	0%	0%	100%
CNN	VH	96,4%	98,4%	7%	1,17%	93%	93,5%	1,64%	7,38%	98,6%
	VH	100%	100%	0%	0%	100%	100%	0%	0%	100%
RNN	VV	96,1%	98,7%	6,5%	1,15%	93,5%	93,9%	1,36%	6,81%	97,3%
	VH	100%	100%	0%	0%	100%	100%	0%	0%	100%
SVM	VV	100%	100%	0%	0%	100%	100%	0%	0%	100%
	VH	100%	100%	0%	0%	100%	100%	0%	0%	100%
Naive Bayes	VV	100%	100%	0%	0%	100%	100%	0%	0%	100%
	VH	100%	100%	0%	0%	100%	100%	0%	0%	100%
Decision Tree	VV	100%	100%	0%	0%	100%	100%	0%	0%	100%
	VH	100%	100%	0%	0%	100%	100%	0%	0%	100%

**Table 9:** The statistical evaluation metric results of all applied models.

Model	Polarization Band	F-1 Score	Specificity	Recall	Precision
Hybrid	VV	0.9997	0.9995	0.9997	0.9997
	VH	1.00	1.00	1.00	1.00
CNN	VV	0.9266	0.9731	0.8865	0.9705
	VH	1.00	1.00	1.00	1.00
RNN	VV	0.9602	0.9192	0.9981	0.9251
	VH	1.00	1.00	1.00	1.00
SVM	VV	1.00	1.00	1.00	1.00
	VH	1.00	1.00	1.00	1.00
Naive Bayes	VV	1.00	1.00	1.00	1.00
	VH	1.00	1.00	1.00	1.00
Decision Tree	VV	1.00	1.00	1.00	1.00
	VH	1.00	1.00	1.00	1.00

From Tables 8 and 9, it is seen that all results for the VH band are at the highest level of success. However, for the VV band deep learning methods seem to have a lower success rate when compared to other methods.

When examining the relationship between SAR backscatter and sea surface object identification at different polarizations, each backscatter value can give different results. For example, [26] recommend HV as it provides the strongest slick sea contrast when the sensor of interest has a sufficiently low noise floor. Otherwise, they found that VV is the most suitable parameter for detecting layers on the ocean surface. For this proposed study, VH backscatter values gave the most optimal result for mucilage areas.

The proposed hybrid Transformer–Logistic Regression model demonstrates high classification performance in detecting marine mucilage over the Armutlu–Zeytinbağı region of the Marmara Sea; however, several limitations should be considered.

The model has undergone specific training and validation using SAR images acquired from a single geographical region. Although the Marmara Sea provides a suitable testbed for mucilage detection due to its history of frequent blooms, applying the same model to different regions or seasons may yield varying results. It is imperative to acknowledge the potential impact of discrepancies in sea surface conditions, anthropogenic activities, and environmental factors on SAR backscatter signatures. These variables have the capacity to exert influence on the observed characteristics of the backscatter signatures, thereby necessitating a comprehensive examination to ascertain their comprehensive impact. Consequently, additional region-specific calibration and external validation would be necessary for generalization.

Despite the utilization of techniques such as data augmentation and 5-fold cross-validation, the relatively limited annotated dataset may incur a risk of overfitting. This is a common challenge in remote sensing applications where labeled data is limited. The expansion of the dataset across a greater number of time periods and a more extensive spatial coverage would contribute to enhancing the robustness and stability of the model.

Sentinel-1’s C-band Synthetic Aperture Radar (SAR) sensors function independently of cloud cover and daylight, thereby offering substantial advantages over optical sensors. However, the reliability of SAR backscatter imaging can be compromised by extreme weather conditions, such as high winds or rough sea states, which can introduce noise and misclassification. Although these effects were mitigated during the preprocessing stage, their potential repercussions must be acknowledged in subsequent operational deployments.

Unlike other optical satellites, synthetic aperture radar (SAR) imagers are active sensors that overcome cloud obscuration, but their capacity to detect macroalgae and other floating matter is generally unknown. In all detection situations, macroalgae features always appear in Sentinel-1/SAR images with positive contrast from the surrounding waters. Therefore, due to the all-weather measurements, SAR observations can complement those from optical sensors in monitoring and detecting object in their respective regions (Qi et al. 2022) [27]. Therefore, backscatter values can always be obtained for VV and VH polarizations, even if it is cloudy.

The results of similar studies are discussed in Table 10.

**Table 10:** The comparison of studies on mucilage fields for detecting object from satellite images.

Dataset	Experimental Area	Accuracy	Methods	Reference
Sentinel-2A & MODIS	Marine Mucilage	90%	Mucilage Index (MI) Formulation	[28]
Sentinel-1-2-3	Marine Mucilage	High Monitoring	Acquisition of In-Situ and Space-Borne Mucilage Data, Water Quality Analyses, And Space-Borne Data Analyses	[12]
Sentinel-2	Marine Mucilage	96%	CNN with NDVI and AMEI Index	[29]
PRISMA Hyperspectral Data	Marine Mucilage	0.0019 (RMSE)	Linear Mixture Model (LMM)	[30]
PRISMA Hyperspectral Data	Marine Mucilage	0.0028 (RMSE)	Generalized-LMM (GLMM)	[31]
PRISMA Hyperspectral Data	Marine Mucilage	0.0019 (RMSE)	Bilinear Mixing Model (BLMM) and Linear-Quadratic Mixing Model (LQMM)	[32]
PRISMA Hyperspectral Data	Marine Mucilage	99%	Nonnegative Matrix Factorization (NMF)-based Unmixing Method	[33]
Sentinel-2	Marine Mucilage	High Monitoring	Random Forest (RF) and CNN for SHAP analyse	[34]
GÖKTÜRK-I	Marine Mucilage	0.0018 (RMSE)	Generalized-LMM (GLMM)	[35]
PRISMA Hyperspectral Data	Marine Mucilage	99%	Multiscale Superpixel-based Nearest Subspace Classifier (MSBNSC)	[36]
Original Taken Some Photos	Marine Mucilage	96%	CNN (ResNet50), kNN, SVM, and FFNN	[10]
Sentinel-2	Marine Mucilage	97%	Vescovi Index + Linear Regression + Random Forest (RF) & U-Net Segmentation	[2]
Sentinel-2 & Landsat-8	Marine Mucilage	99%	Random Forest (RF)	[13]
Sentinel-2	Marine Mucilage	99.9%	Random Forest (RF)	[1]
Google Earth Pro Satellite Images	Marine Mucilage	100%	CNN Deep Learning	[37]
Sentinel-2	Marine Mucilage	>90%	Random Forest (RF)	[38]
GNSS satellite signals (e.g., GPS, GLONASS)	Precise Point Positioning (GNSS satellite navigation)	High (not specified)	Factor Graph Optimization	[39]
Planetary images from planetary missions (e.g., Lunar Reconnaissance Orbiter, Mars Reconnaissance Orbiter)	Planet Crater Detection	~85%+ (not specified precisely)	Unsupervised Domain Adaptation	[40]
Images captured under low light from various terrestrial sensors or satellites	Low-Light Image Enhancement	PSNR approx. 30–35 (estimated)	Dehazing Physical Model Based Enhancement	[41]
Multi-view image datasets (possibly from multiple camera systems or multi-angle satellites)	Multi-View Classification	90%+ (not specified precisely)	GCCNet (Gated Cross-Correlation Network)	[42]
Molecular datasets (chemical databases, simulation data)	Molecular Property Prediction	Improved accuracy	Multi-view Molecular Representation Learning (MvMRL)	[43]
Remote sensing data from global inland water bodies (likely Sentinel-2, Landsat, MODIS)	Phycocyanin Monitoring via Remote Sensing	High (not specified)	Remote Sensing, Spectral Analysis	[44]
Spaceborne SAR data (e.g., Sentinel-1 SAR)	Wave Attenuation by Sea Ice	High model accuracy	Spaceborne SAR Analysis	[45]
Hazy images from terrestrial or satellite sensors (possibly from urban or natural scenes)	Fast Image Dehazing	PSNR approx. 28–33	Linear Transformation Based Dehazing Method	[46]
Various images (not specifically satellite; possibly benchmark datasets)	Image Feature Extraction	High success rate	Convolution-Transformer Hybrid Model	[47]
Optical and ISAR images from radar and optical satellite sensors	Image Translation and Domain Adaptation	High (not specified)	Meta-Learning Based Domain Prior	[48]
Marine biology images (likely underwater cameras or remote sensing)	Enhanced Visual Perception of Marine Biology Images	High (not specified)	Diffusion-GAN	[49]
Remote sensing hazy images (likely satellite images, e.g., Sentinel-2, Landsat)	Image Dehazing	High PSNR (not specified)	Wavelet-based Generative Adversarial Networks	[50]
Marine imagery datasets (underwater or surface, possibly drone or satellite images)	Marine Biodetection	High accuracy	Improved YOLOv10 (Lightweight Model)	[51]
**Sentinel-1**	**Marine Mucilage**	**100%**	**Hybrid Model (Transformer Encoder + Logistic Regression), Deep Learning (RNN, CNN) and Machine Learning Models (Decision Tree, Naive Bayes, SVM)**	**Proposed Approach**

## 4. Conclusion

Remote sensing systems are becoming more important day by day and these are systems that are frequently used in many areas and make life significantly easier. Therefore, today, remote sensing systems are in an extremely important position. In this study, using a remote sensing system, it is presented to investigate the automatic detection of mucilage areas that are dangerous for marine life, create visual pollution and cause bad odor.

The work consists of several stages. In the first stage, mucilage areas that occurred to a large extent in May 2021 were determined. This mucilage areas determined in the second stage were marked manually on the google earth engine code platform. Then, these marked points were transferred to Sentinel-1 satellite data by using time series over Google Earth Engine Code platform with the help of GPS. Then, the backscattering values of the two polarization bands (VV, VH) connected to the Sentinel-1 satellite were taken for each point via the Google earth engine code platform, a data set in two bands was created. The dataset includes mucilage field data for 17 May 2021 and 22 May 2021, and clean water field data for 21 June 2021 and 22 June 2022. In the last stage, it is aimed to automatically detect mucilage areas by applying deep learning and machine learning models to these data sets. It is important to take the value at each point marked in the mucilage area

(Armutlu-Zeytinbağı) from the same point after 1 month in order to determine the mucilage area. As can be seen in Fig 3 and Fig 4, the distribution of data sets shifts after a certain point. The first 1300 samples are on the dates when there is mucilage, and the last 1300 samples are on the dates when there is no mucilage. Therefore, in the data distribution, the data were cut from the middle point and displaced. This proof that the marked points are the mucilage zone on May 17 and May 22 and the clean water zone on June 21 and June 22.

The datasets used in the study have 2 attributes and 2 classes. The attributes were determined as May 2021, when mucilage occurs most frequently, and June 2021, when mucilage loses its effect. The reason why the attributes are negative is because the backscattering property of water is lower than other objects.

Mucilage has a very serious impact on the marine ecosystem. It reduces the oxygen level in the sea, harms sea creatures, restricts fishing activities, creates visual pollution and kills bottom plants that feed the sea. Therefore, it is important to identify the mucilage areas and to take the necessary measures after detection.

The study can be used in the future to autonomously receive and analyze satellite data instantaneously and as a result, to detect mucilage areas. Considering all these, the proposed study has an important place directly in the marine ecosystem and indirectly human ecosystem.

Considering the results, it is seen that the lowest results are the deep learning methods used for the VV band. This may seem to be due to the insufficient number of data used for deep learning. However, when the same number of data is used for the VH band, the results are excellent. The reason why the VV band gives low results may be that the VV band receives point data while receiving data. As a result, the success of the applied models is very high. Therefore, this high success leads to the conclusion that the used models can also be used in the automatic detection of mucilage systems. In addition, when the data obtained are examined, the results of deep learning and machine learning models belonging to both VV band and VH band have very high accuracy values (96%−100%) is seen to have. Therefore, the mucilage area can be detected automatically by using deep learning and machine learning methods.

This study successfully demonstrated the high potential of advanced machine learning and hybrid modelling approaches for the detection of mucilage areas using Sentinel-1 satellite data. By capitalising on the distinctive capabilities of RNN, CNN, SVM, decision tree, Naive Bayes, and a pioneering Transformer-Logistic Regression hybrid model, we attained an exceptional level of classification accuracy, reaching 100%. This exceptional outcome serves to reinforce the robustness and precision of our methodology, particularly in the context of handling complex spatial and spectral patterns in satellite imagery. The combination of the Transformer model, which is adept at capturing long-range dependencies, with logistic regression yielded an effective equilibrium between interpretability and performance. These results demonstrate the feasibility and effectiveness of our approach in addressing real-world environmental challenges, thereby paving the way for more reliable and scalable monitoring of marine ecosystems.

In this study, we developed a hybrid deep learning framework combining a Transformer-based feature extractor with Logistic Regression for automatic detection of marine mucilage using Sentinel-1 SAR data, specifically applied to the Armutlu–Zeytinbağı region of the Marmara Sea. The model exhibited high accuracy and robustness in distinguishing mucilage presence from background SAR signals.

For subsequent research endeavors, there are several promising avenues that merit exploration. Firstly, the integration of real-time satellite data streams has the potential to facilitate near-real-time monitoring of mucilage spread, thereby supporting the development of early warning systems for coastal management. Secondly, the proposed methodology can be adapted and tested for the detection of other marine environmental hazards, such as oil spills and harmful algal blooms, given the shared characteristics in SAR backscatter behavior. A multi-sensor data fusion approach, integrating Sentinel-1 SAR with Sentinel-2 optical imagery, has the potential to enhance spatial and spectral resolution, thereby improving the accuracy and interpretability of classification tasks.

## Supporting information

S1 Data Datasets. (RAR)
